# Efficacy of acupuncture for whiplash injury: A protocol for systematic review and meta-analysis

**DOI:** 10.1097/MD.0000000000027767

**Published:** 2021-12-10

**Authors:** Sang-Hyun Lee, Hye-Jin Park, Hyun-Tae Kim, Sun-Young Park, In Heo, Eui-Hyoung Hwang, Byung-Cheul Shin, Man-Suk Hwang

**Affiliations:** a Department of Korean Medicine Rehabilitation, Spine and Joint Center, Pusan National University Korean Medicine Hospital, Yangsan, Republic of Korea; b 3rd Division of Clinical Medicine, School of Korean Medicine, Pusan National University, Yangsan, Republic of Korea.

**Keywords:** acupuncture, electro-acupuncture, protocol, randomized controlled trial, systematic review, whiplash-associated disease

## Abstract

**Background:**

Studies in both Eastern & Western countries such as the United States and Europe have evaluated the efficacy of acupuncture for whiplash injury or whiplash-associated disorder (WAD). However, no systematic reviews on the effectiveness of acupuncture on WAD have been conducted since 2014. Therefore, we are planning an updated systematic review of studies published since 2014 to overcome the limitations of existing evidence.

**Methods:**

Literature will be identified from searches of relevant databases, including international databases such as PubMed, Ovid-Medline, Embase, The Cochrane Library, and China National Knowledge Infrastructure and Korean databases such as Korea Med, Korean Studies Information Service System, Oriental Medicine Advanced Searching Integrated System, and National Digital Science Library. Only randomized controlled trials using acupuncture or electro-acupuncture for whiplash injury will be included. The primary outcomes will be the visual analog scale or numerical rating scale of the neck pain, while the secondary outcome is the range of motion of the neck. The risk of bias for individual papers will be assessed by two independent investigators using the Cochrane “Risk of Bias” assessment tool.

**Dissemination:**

We plan to report the results of the study in a peer-reviewed journal after completing the research. In addition, we expect this study to provide invaluable information to clinicians treating patients with WAD with acupuncture or electro-acupuncture.

**Trial registration number:**

PROSPERO 2021: CRD42021261595. Registered on 18 July 2021. https://www.crd.york.ac.uk/prospero/display_record.php?RecordID=261595

## 1 Introduction

According to WHO statistics, the number of road traffic deaths has increased in the last decade, with 1.35 million reported in 2018. In addition, road traffic injuries are the eighth highest cause of death for all age groups, which is higher than HIV/AIDS, tuberculosis diseases.^[1]^

Whiplash injury or whiplash-associated disorder (WAD) occurs when the energy of the acceleration-deceleration mechanism in a car collision shifts to neck.^[2]^ Excessive neck extension and flexion lead to neck pain and headache, which are the chief complaints of WAD.^[3]^ Acute and subacute WAD generally receive conservative treatments with active mobilization rather than passive treatment.^[4]^

In Korea, traditional Korean medicine including acupuncture is commonly used for the treatment of WAD to control the pain and dysfunction. Recent trials not only in Eastern countries such as Korea and China but also in Western countries such as the United States and Europe have assessed the efficacy of acupuncture for WAD. Sterling et al,^[5]^ Tobbackx et al,^[6]^ and Tough et al^[7]^ conducted randomized controlled trials (RCTs) in Australia, Belgium, and the UK, respectively. However, no study has systematically examined the effectiveness of acupuncture on WAD since the study by Moon et al^[8]^ in 2014. While Lee et al^[9]^ reported a protocol for the systematic review of acupuncture on WAD, they did not report the results of a subsequent study.

Therefore, we are planning an updated systematic review of acupuncture and electro-acupuncture for WAD including RCTs published after the systematic review by Moon et al^[8]^ in 2014 and overcoming the limitations of existing evidence.

## 2 Methods and analysis

### 2.1 Eligibility criteria

The eligibility criteria are RCTs assessing the efficacy of acupuncture and electro-acupuncture on WAD, regardless of the reporting type, blinding, and language. The population will include WAD patients defined or specified by any diagnostic criteria, regardless of their race, age, and sex. Non-randomized controlled trials, single-arm pre- and post-clinical trials, case-control studies, case reports, laboratory studies (including in vivo and in vitro), letters, and reviews will be excluded. The interventions in the experimental group will be acupuncture or electro-acupuncture, and RCTs comparing acupuncture combined with active treatment(s) to the same active treatment(s) will also be included. The control group intervention is usual care, including physiotherapy, medications, conventional treatments other than acupuncture, and sham treatment. The primary outcomes are visual analog scale or numeral rating scale scores of the neck pain, while the secondary outcomes will be the range of motion of the neck and the safety.

### 2.2 Search methods for study identification

The search will begin on October 1, 2021, in the following databases: non-Korean databases such as PubMed, Ovid-Medline, Embase, The Cochrane Library, and China National Knowledge Infrastructure and Korean databases such as Korea Med, Korean Studies Information Service System, National Digital Science Library, and Oriental Medicine Advanced Searching Integrated System. The publication years will be from the inception of each database to present. We will provide search terms for the representative databases for each language in English, Chinese, and Korean in the Appendix. In addition, to identify studies other than those identified in the database searches, we will also review the references of previously published conferences and studies.

### 2.3 Method of data collection and analysis

#### 2.3.1 Study selection

**Figure 1:**
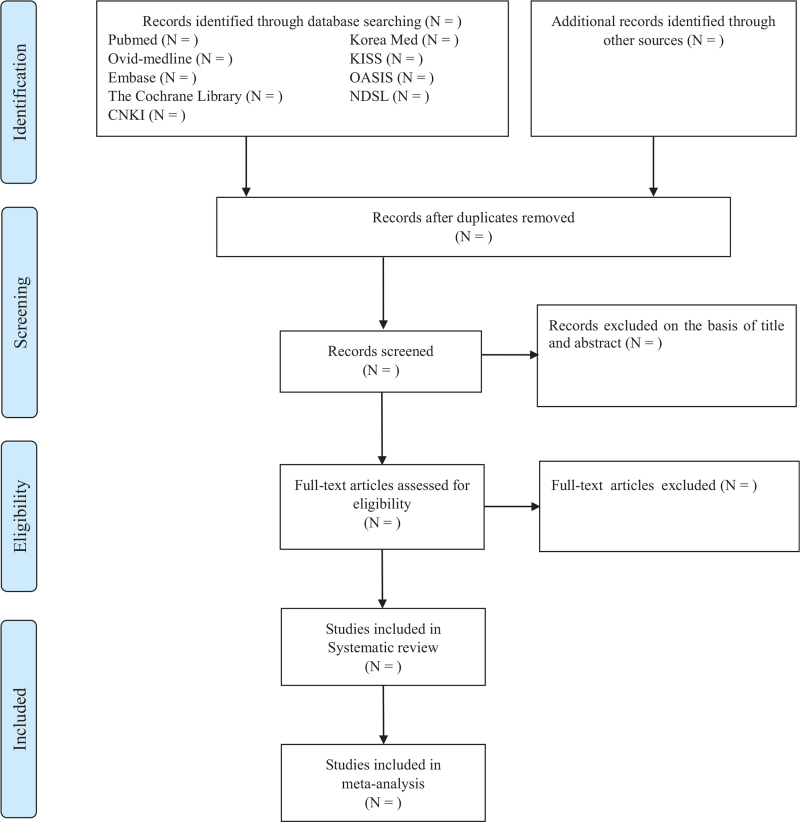
PRISMA flowchart template for the included studies.

Only RCTs using acupuncture or electro-acupuncture for whiplash injury will be included. We will organize the retrieved studies using Endnote 20 (Clarivate Analytics, London, UK). First, after removing duplications, we will exclude studies that do not meet the eligibility criteria based on screening of the titles and abstracts. Subsequently, two independent investigators will thoroughly review the full text of the individual studies. Disagreements will be resolved through discussion between the two investigators, with arbitration by a consultation third reviewer if an agreement is not reached. The detailed procedure for study selection will be displayed as a PRISMA flowchart (Fig. 1), with the reasons for study exclusion separately arranged in a table.

#### 2.3.2 Data extraction and management

After excluding the literature based on title and abstract review, two independent investigators will verify the full texts of the individual papers to extract, analyze, and tabulate data on study design, blinding, applied interventions, control group interventions, outcomes, and other details will be. The study authors will be contacted for further data, if necessary.

#### 2.3.3 Risk of bias

Two independent investigators will determine the risk of bias for individual papers using the Cochrane collaboration “Risk of Bias” tool. This assessment tool considers random sequence generation, allocation concealment, blinding of participants and personnel, blinding of outcome assessment, incomplete outcome data, selective reporting, and other sources of bias. Each bias will be classified as high, low, or unclear.^[10]^

#### 2.3.4 Management of missing data

In case of insufficient or missing data, we plan to contact the author through email or phone. If enough data are not obtained, we will not include the study. The authors discuss the potential influence of insufficient or missing data.

#### 2.3.5 Measures of treatment effect

Relative risk is the main tool used for dichotomous data to measure the treatment effect. Standard mean differences or mean differences are the main tools for continuous data. All data, including dichotomous and continuous data, will be presented with 95% confidence intervals.

#### 2.3.6 Synthesis of data

RevMan version 5.4 (Cochrane) software is the main tool for meta-analysis. To synthesize the data, a fixed-effects model will be used in the case of no statistical heterogeneity. Otherwise, in case of significant statistical heterogeneity, further analysis will be performed to determine the reasons for the heterogeneity. In cases of evident clinical heterogeneity, the study will be excluded, and then a meta-analysis will be performed using a random-effects model.

#### 2.3.7 Subgroup analysis

In cases of significant heterogeneity during clinical research, subgroup analysis will be performed. The subgroup criteria may include the needle insertion time, number of treatments, control group intervention, severity of whiplash injury, and etc.

#### 2.3.8 Sensitivity analysis

In a sufficient number of studies are identified, the robustness of the results will be assessed in a sensitivity analysis. The key criteria are the method quality, number of samples, and missing data selection.^[11]^

#### 2.3.9 Heterogeneity assessment

Heterogeneity will be assessed using chi-square tests and represented by the *I*^2^ value. An *I*^2^ value of <50% indicates negligible heterogeneity and a fixed-effects model will be used to estimate the effect size. An *I*^2^ value exceeding 50% indicates the presence of heterogeneity between studies and subgroup analysis will be performed to determine the cause of heterogeneity.

#### 2.3.10 Reporting bias

If more than 10 studies are included, funnel plots will be generated to examine publication bias.^[12]^ In general, large numbers of subjects show a small range of effect sizes due to high accuracy, while a small number of subjects show a large range of effect sizes due to low accuracy. No publication bias should appear in the funnel plot. Egger regression tests^[13]^ will be used as an alternative tool to check the asymmetry of the funnel plot based on the *P* value.

#### 2.3.11 Evidence quality grading

The Grading of Recommendations Assessment, Development and Evaluation method will be applied to categorize the evidence quality of the outcomes as high, moderate, low, or very low.

### 2.4 Ethical statement and dissemination

As this study aims to perform a systematic review based on published papers, ethical approval or patient consent is not required. We plan to report the results of the study in a peer-reviewed journal after completing the research.

## 3 Discussion

Moon et al^[8]^ previously conducted a systematic review of acupuncture or electro-acupuncture for whiplash injury in 2014. Their study included papers published up to October 2013. Only six studies were included, and the statistical and clinical heterogeneity made the quality of the studies implausible. The present study will include more studies of higher quality and additional studies published after October 2013 using a broad search strategy.

The planned systematic review will evaluate the efficacy and safety of acupuncture and electro-acupuncture for whiplash injury. The predicted limitation of this study is that it has language limitations; namely, English, Chinese, and Korean. We expect this study to provide invaluable information to clinicians treating patients with WADs with acupuncture or electro-acupuncture.

## Author contributions

**Conceptualization:** Sang-Hyun Lee.

**Investigation:** Sang-Hyun Lee, Hye-Jin Park, Hyun-Tae Kim, Sun-Young Park.

**Methodology:** In Heo, Byung-Cheul Shin.

**Project administration:** Eui-Hyoung Hwang, Man-Suk Hwang.

**Supervision:** Man-Suk Hwang.

**Writing – original draft:** Sang-Hyun Lee.

**Writing – review & editing:** Sang-Hyun Lee, Byung-Cheul Shin, Man-Suk Hwang.

## Supplementary Material

Supplemental Digital Content
