# Supplementary material for: Efficacy of acupuncture for whiplash injury: A protocol for systematic review and meta-analysis
Source: Medicine (Baltimore). 2021 Dec 10;100(49):e27767. doi: 10.1097/MD.0000000000027767 (PMC8663840; doi:10.1097/MD.0000000000027767)
Supplement: Supplemental Digital Content [file medi-100-e27767-s001.docx]

Appendix Ⅰ. Search strategy used in representative databases.

(1) Pubmed

#1 whiplash OR acute whiplash injury* OR acute whiplash associated disorder* OR acute WAD OR acute whiplash associated disorder* II OR acute WAD II OR whiplash associated disorder* OR WAD OR whiplash associated disorder* II OR WAD II, OR whiplash OR whiplash injury* OR whiplash patient* OR whiplash syndrome* OR cervical spine disorder* OR cervical spine injury*) OR "Accidents, Traffic" [Mesh] OR (("Motor Vehicles"[Mesh:NoExp] OR "Automobiles"[Mesh] OR "Motorcycles"[Mesh] OR traffic[tiab] OR vehicle[tiab] OR vehicular[tiab] OR car[tiab] OR cars[tiab] OR automobile[tiab] OR automobiles[tiab] OR motorcycle[tiab] OR motorcycles[tiab] OR taxi[tiab] OR cab[tiab] OR road[tiab] OR pedestrian[tiab] OR pedestrians[tiab]) AND (accident[tiab] OR accidents[tiab] OR injury[tiab] OR injuries[tiab] OR crash[tiab] OR crashes[tiab] OR "Wounds and Injuries"[Mesh] OR "injuries"[Subheading])) AND (cervic* OR thoracic* OR lumba*)

#2 acupuncture

#3 electroacupuncture

#4 acupressure

#5 ((((((((((((((((((((((((meridian) OR acupoint) OR acupuncture [mh]) OR acupuncture Analgesia [mh]) OR acupuncture Therapy [mh]) OR acupuncture points [mh]) OR acupuncture, ear [mh]) OR acupuncture [Text Word]) OR acupressure [Text Word]) OR electroacupuncture) OR electro acupuncture) OR electro-acupuncture) OR meridian* [Text Word]) OR needling [Text Word]) OR acup-point*) OR acu point* [Text Word]) OR acupoint* [Text Word]) OR Acupuncture [mh]) OR electroacupuncture [mh]) OR acupuncture* [Text Word]) OR elctroacupuncture* [Text Word]) OR (acupuncture AND th[sh])) OR acupuncture[tiab]) OR acupuncture[mh]) OR acupuncture/th[mh]

#6 #2 or #3 or #4 or #5

#7 #1 and #6

(2) China National Knowledge Infrastructure (CNKI)

1 (SU='traffic' OR SU='交通事故') AND (SU='骨折' or SU='fracture') AND (SU='acupuncture' OR SU='針' or SU='electro acupuncture' OR SU='电針' or SU='meridian' OR SU='经穴' or SU='acupoint' or SU='acupuncture-ear' OR SU='耳针')

(3) Korea Med

1 (traffic [ALL] or automobile [ALL]) AND fracture [ALL] AND acupuncture [ALL] AND electro-acupuncture [ALL] AND merdian[ALL] AND acupoint [ALL])
